# Relationship Between Gestational Weight Gain and Pregnancy Complications or Delivery Outcome

**DOI:** 10.1038/s41598-017-12921-3

**Published:** 2017-10-02

**Authors:** Wenjia Yang, Feifei Han, Xueying Gao, Yifei Chen, Linong Ji, Xiaoling Cai

**Affiliations:** Endocrinology and Metabolism Department, Peking University People’s Hospital, No. 11 Xizhimen South Street, Xicheng District, Beijing P.R. China

## Abstract

The purpose of this study is to analyse the association between gestational weight gain and delivery outcome or the morbidity of pregnancy complications. A total of 1,102 pregnant women who delivered at Peking University People’s Hospital in China between January 2011 and December 2012 were included in this study. We divided them into 4 groups according to the baseline BMI quartiles and weight gain quartiles in different trimesters of pregnancy to analyse the status of delivery outcome and morbidity of pregnancy complications. Baseline BMI was significantly positive correlated with the morbidity of gestational hypertension and gestational diabetes. Weight gain in the second trimester of pregnancy was significantly positively correlated with the morbidity of macrosomia. Weight gain in the third trimester of pregnancy showed significantly positive correlation with the morbidity of macrosomia, and significantly negative correlation with the morbidity of neonatal death, preterm birth, gestational diabetes, and low birth-weight infant. Gestational weight gain showed significantly positive correlation with the morbidity of macrosomia and significantly negative correlation with neonatal death, stillbirth, gestational diabetes, preterm birth and low birth-weight infant. There is a correlation between baseline BMI, pregnancy weight gain and gestational complications, adverse pregnancy outcomes, and status of neonate in varying degrees.

## Introduction

In China, inadequate body weight control has become a prominent health problem with changes in lifestyle. For pregnant women who are in a special physiological condition^1^, appropriate pre-pregnancy weight and gestational weight gain (GWG) to meet the demands of mother and foetus is essential. Previous studies showed increasing pre-pregnant body mass index (BMI) was associated with increasing risk of gestational hypertension and gestational diabetes mellitus (GDM)^2–5^. Women who are overweight or obese before pregnancy are subject to increased risk of macrosomia and dystocia^6–8^. Women who are underweight were less likely to have adverse pregnancy outcomes, but more likely to have intrauterine growth restricted infants^3^.

GWG also affects obstetrical and neonatal outcomes. Several studies suggest associations between excessive GWG and risk of hypertension, macrosomia, and caesarean section in pregnant women of different ethnics groups^9–13^. Inadequate GWG increased the risk of preterm birth and growth-restricted infants^10–12^. However, thus far, few studies have explored appropriate weight gain in different trimesters during pregnancy.

In 2009, the Institute of Medicine (IOM) provided specific recommendations regarding the ideal gestational weight gain according to the BMI categories^14^. However, the report was criticized by some health care providers saying that the recommendation was still too high for the obese women. In Japan, as there are more underweight women, the obesity classification is different, so the recommendation issued by the Japanese Ministry of Health, Labour and Welfare on gestational weigh gain differs from that developed by IOM^15^. In China, we have been following the recommendation of IOM and there is no established recommendation for Chinese women.

Because established recommendations on weight gain during pregnancy are inconsistent and contentious, ethnics groups, national conditions and many other factors should be taken into account for the recommendations, the aim of this study is to analyse the relevance between baseline BMI, gestational weight gain, and delivery outcome in a Chinese population.

## Methods

### Populations

All women who delivered at Peking University People’s Hospital in China between January 2011 and December 2012 were considered for inclusion. Inclusion criteria were women who had regular prenatal care and maintained complete data of prenatal care in Peking University People’s Hospital during 2011-2012. Exclusion criteria were women with insufficient information about their gestational weight during the pregnancy; multiple pregnancy. In all, 1,102 women were eligible for the final analysis.

Data of women studied were extracted from Peking University People’s Hospital electronic medical records. The study was approved by the ethics committee of Peking University People’s Hospital. All of the health care procedures were carried out in accordance to the approved guidelines and regulations. Informed consent was obtained from all participants.

### Data collection and definitions of variables

The following data were collected from electronic medical records for each pregnant woman: maternal sociodemographic characteristics (age at delivery, height, parity), lifestyle behaviours (smoking and alcohol use), maternal weight at the 12^th^ and 28^th^ weeks of pregnancy and weight at delivery, laboratory and physical examination during the first prenatal care [alanine transaminase (ALT), aspartate aminotransferase (AST), serum creatinine (Scr), total cholesterol (TC), low-density lipoprotein (LDL-C), high-density lipoprotein (HDL-C), triglyceride (TG), uric acid (UA), fasting glucose, hemoglobin, systolic blood pressure, diastolic blood pressure], adverse pregnancy outcomes (abortion, induction of labour, neonatal death, stillbirth, preterm birth), accessory abnormality (abnormality of umbilical cord, amniotic fluid and placenta), obstetric complications (puerperal infection, dystocia), foetal abnormality (foetal distress, macrosomia, low birth-weight infant, foetal anomaly), complications of gestation [gestational diabetes mellitus (GDM), gestational hypertension, preeclampsia/eclampsia].

Abortion in this study included threatened abortion and spontaneous abortion before 28^th^ week with foetus weight less than 1,000 g. Neonatal death included early neonatal deaths or death of a baby within seven days of extra uterine life and deaths that occur after the first week of extrauterine life but within the first 28 days after birth. Preterm birth was defined as a birth before 37 weeks’ gestation. Stillbirth was defined as the *in-utero* death of a baby after 24 completed weeks’ gestation. Abnormality of umbilical cord included prolapse of umbilical cord, entanglement cord, umbilical cord knot and length abnormality (length < 30 cm or >80 cm). Abnormality of amniotic fluid included polyhydramnios (maximal deepest pocket ≥ 8 cm and/or amniotic fluid index ≥ 20 cm) and oligohydramnios(amniotic fluid index < 5 cm and/or maximal deepest pocket < 2 cm). Abnormality of placenta in our study referred to placental abruption, which was defined as the abnormal separation after 20 weeks of gestation and prior to birth. Puerperal infection was defined as genital tract infection in puerperium. Dystocia referred to difficult labour characterized by prolonged labour. Foetal distress was diagnosed as follows: (1) the meconium-stained amniotic fluid reached the second or third degree; (2) auscultate foetal heart rate twice or more during the contraction breaks, foetal heart rate was greater than 160 beats per minute or less than 120 beats per minute; (3) foetal heart electric monitor slows down, and variable deceleration appears frequently. The non-stress test baseline rate is abnormal, with decreased or no variation, or no response; (4) foetal movements less than three times per hour. Macrosomia was defined as a neonatal birthweight 4,000 g or more. Low birth-weight defined as a neonatal birthweight 2,500 g or less after 37 weeks. GDM (by 75-g oral glucose tolerance test) was diagnosed when at least one of the following was found: fasting blood glucose level of ≥92 mg/dL, blood glucose level at 1 hour of ≥180 mg/dL, blood glucose level at 2 hour ≥ 153 mg/dL during 24–28 weeks. Gestational hypertension was defined as a case in which hypertension (systolic blood pressure ≥ 140 mmHg and/or diastolic blood pressure ≥ 90 mmHg) developed after 20 weeks of gestation. Preeclampsia was defined by the onset of hypertension (≥140/90 mmHg) and proteinuria after 20 weeks of gestation in a previously normotensive woman. Eclampsia was defined as the occurrence of convulsions and/or coma unrelated to other cerebral conditions in women with signs and symptoms of preeclampsia.

The prenatal care records did not contain data on maternal weight before pregnancy, and body weight at 12^th^ week was the first recorded weight, hence, we used the mother’s weight at 12^th^ weeks as the first trimester weight to calculate baseline BMI. Weight at 28^th^ week and 1 week prior to delivery was defined as second and third trimester weight, respectively. Weight gain during the second trimester was defined as the difference between first trimester and second trimester. Weight gain during the third trimester was defined as the difference between second trimester and third trimester.

Maternal baseline BMI was categorized as underweight group (BMI < 18.5 kg/m^2^), normal weight group (18.5 ≤ BMI < 24.0 kg/m^2^), overweight group (24.0 ≤ BMI < 28.0 kg/m^2^) and obesity group (BMI ≥ 28.0 kg/m^2^) according to the “Guideline of controlling and prevention on overweight and obesity in China”^16^. Baseline BMI was also classified by WHO definition as underweight group (BMI < 18.5 kg/m^2^), normal weight group (18.5 ≤ BMI < 25.0 kg/m^2^), overweight group (25.0 ≤ BMI < 29.0 kg/m^2^) and obesity group (BMI ≥ 30.0 kg/m^2^)^14^. We further divided them into 4 groups according to weight gain quartiles in different trimesters of pregnancy.

### Statistically analysis

SPSS version 19.0 was used for statistical analyses. Continuous data were expressed as mean ± standard deviation if normally distributed. Categorical data were expressed as percentages. To compare variables between the different groups, an independent sample t-test was used for normally distributed continuous variables. Chi-squared tests were used for categorical variables. All P values were two-sided, and P values less than 0.05 were considered to indicate statistical significance. Binary logistic regression analysis and multivariable logistic regression analysis were used to assess the association between the baseline BMI and delivery outcome or pregnancy complications adjusting for confounding variables, including age, serum glucose, blood pressure, smoking and alcohol use, Scr, liver function and level of lipid, which showed significant difference in the independent sample t-test. Association between weight gain in different trimesters and delivery outcome or pregnancy complication was also analysed.

## Results

### Characteristics of included pregnant women

A total of 1,102 pregnant women were included in this analysis. The average age of delivery for this population was 30.4 ± 3.3 years and baseline BMI was 21.6 ± 3.1 kg/m^2^. Table 1 shows grouping according to quartile for baseline BMI, weight in the first trimester, weight gain in the second trimester, weight gain in the third trimester, and GWG.

**Table 1:** Baseline characteristics of pregnant women categorized by quartile for baseline BMI, weight in the first trimester, weight gain in the second trimester, weight gain in the third trimester, and GWG.

	1^st^ Quartile	2^nd^ Quartile	3^rd^ Quartile	4^th^ Quartile
**Baseline BMI Quartile Category**				
Baseline BMI(kg/m^2^)	18.1 ± 1.0	20.3 ± 0.5	22.1 ± 0.6	25.8 ± 2.4
Age(years)	29.5 ± 3.2	30.4 ± 3.3	30.7 ± 3.2	31.2 ± 3.5
Fasting glucose(mmol/L)	4.3 ± 0.5	4.3 ± 0.4	4.3 ± 0.4	4.4 ± 0.8
SBP(mmHg)	107.6 ± 11.2	110.5 ± 10.3	112.5 ± 11.0	116.2 ± 12.1
DBP(mmHg)	69.8 ± 8.3	70.8 ± 8.1	71.9 ± 8.9	74.5 ± 8.5
ALT(U/L)	17.5 ± 5.5	16.0 ± 18.0	17.5 ± 12.8	21.0 ± 23.2
AST(U/L)	16.0 ± 7.2	18.1 ± 6.8	18.6 ± 6.2	18.9 ± 10.6
LDL-C(mmol/L)	1.99 ± 0.40	2.26 ± 0.57	2.21 ± 0.53	2.45 ± 0.58
HDL-C(mmol/L)	1.63 ± 0.29	1.65 ± 0.28	1.61 ± 0.41	1.53 ± 0.32
CRE(μmol/L)	43.3 ± 6.5	44.7 ± 6.2	45.3 ± 6.9	44.7 ± 6.0
**Weight Gain in the Second Trimester Quartile Category**				
weight gain in the second trimester(kg)	−3.0–6.9	7.0–8.9	9.0–10.5	11.0–21.0
Age(years)	31.2 ± 3.6	30.3 ± 3.2	30.6 ± 3.2	29.9 ± 3.4
Fasting glucose(mmol/L)	4.3 ± 0.5	4.3 ± 0.4	4.3 ± 0.4	4.4 ± 8.3
SBP(mmHg)	113.2 ± 17.3	112.2 ± 11.6	110.0 ± 11.4	111.0 ± 10.7
DBP(mmHg)	72.1 ± 10.9	72.4 ± 8.8	71.0 ± 8.8	71.6 ± 8.1
ALT(U/L)	17.7 ± 18.3	15.4 ± 16.2	17.7 ± 18.9	19.0 ± 21.4
AST(U/L)	18.3 ± 8.7	17.6 ± 7.0	18.5 ± 7.1	19.2 ± 9.2
LDL-C(mmol/L)	2.36 ± 0.63	2.22 ± 0.53	2.17 ± 0.53	2.20 ± 0.52
HDL-C(mmol/L)	1.55 ± 0.32	1.56 ± 0.41	1.59 ± 0.28	1.67 ± 0.32
CRE(μmol/L)	44.2 ± 7.3	44.5 ± 5.7	44.7 ± 6.3	44.7 ± 6.8
**Weight Gain in the Third Trimester Quartile Category**				
weight gain in the third trimester(kg/m^2^)	−2.3–3.6	4.0–5.9	6.0–7.8	7.9–15.0
Age(years)	30.7 ± 3.6	30.8 ± 3.3	30.2 ± 3.2	29.8 ± 3.2
Fasting glucose(mmol/L)	4.3 ± 0.4	4.3 ± 0.4	4.3 ± 0.4	4.4 ± 0.8
SBP(mmHg)	113.0 ± 12.4	112.8 ± 12.0	112.0 ± 10.4	110.2 ± 11.9
DBP(mmHg)	72.6 ± 9.6	72.1 ± 9.1	71.8 ± 7.9	71.0 ± 8.7
ALT(U/L)	18.5 ± 16.1	17.5 ± 20.0	15.3 ± 10.6	17.7 ± 18.9
AST(U/L)	18.7 ± 7.4	18.4 ± 9.1	17.7 ± 6.3	18.5 ± 7.1
LDL-C(mmol/L)	2.44 ± 0.67	2.24 ± 0.50	2.17 ± 0.53	2.16 ± 0.54
HDL-C(mmol/L)	1.57 ± 0.32	1.59 ± 0.32	1.59 ± 0.28	1.61 ± 0.32
CRE(μmol/L)	44.1 ± 6.5	44.6 ± 5.9	44.4 ± 7.1	44.7 ± 6.3
**Gestational Weight Gain Quartile Category**				
GWG(kg)	1.0–11.8	12.0–14.6	15.0–17.7	18.0–29.0
Age(years)	31.2 ± 3.6	30.8 ± 3.1	30.2 ± 3.2	29.8 ± 3.4
Fasting glucose(mmol/L)	4.3 ± 0.4	4.3 ± 0.4	4.3 ± 0.4	4.4 ± 0.8
SBP(mmHg)	114.5 ± 12.2	116.7 ± 9.8	118.5 ± 10.8	119.2 ± 10.3
DBP(mmHg)	73.4 ± 8.7	74.8 ± 7.9	75.9 ± 8.7	75.0 ± 8.7
ALT(U/L)	18.1 ± 16.3	15.6 ± 14.2	17.8 ± 21.5	17.0 ± 14.2
AST(U/L)	18.5 ± 8.3	17.8 ± 6.7	18.4 ± 8.8	18.3 ± 6.3
LDL-C(mmol/L)	2.40 ± 0.66	2.19 ± 0.56	2.20 ± 0.42	2.17 ± 0.56
HDL-C(mmol/L)	1.54 ± 0.34	1.58 ± 0.38	1.62 ± 0.29	1.64 ± 0.33
CRE(μmol/L)	43.9 ± 6.6	44.8 ± 6.8	44.3 ± 6.0	44.7 ± 6.8

Among 1,102 women, 214 (19.4%) women developed adverse pregnancy outcomes, including abortion (15.4%), induction of labour (0.5%), neonatal death (0.7%), stillbirth (0.4%), and preterm birth (4.9%); 480 (43.6%) women developed accessory abnormality, including abnormality of umbilical cord (32.0%), amniotic fluid (13.2%), and placenta (0.6%); 28 (2.4%) women developed obstetric complications, including dystocia (1.3%), and puerperal infection (1.3%); 244 (22.1%) foetuses developed foetal abnormality, including foetal distress (10.6%), macrosomia (7.5%), low birth-weight infant (4.4%), and foetal anomaly (1.1%); 201 (18.2%) women developed gestational complications, including GDM (13.1%), gestational hypertension (5.0%), and preeclampsia/eclampsia (1.9%).

### Correlation between baseline BMI, weight gain in the second trimester, weight gain in the third trimester, GWG and pregnancy complications and delivery outcomes

Weight gain in the second trimester (P < 0.001=, GWG (P < 0.001=, neonate weight (P = 0.002), development of macrosomia (P = 0.029), morbidity of GDM (P = 0.017) and gestational hypertension (P < 0.001= significantly differed across the baseline BMI groups categorized by the “Guideline of controlling and prevention on overweight and obesity in China.” No significant association was found between baseline BMI and adverse pregnancy outcomes, accessory abnormality or obstetric complications. (Table 2) The analysis based on baseline BMI categorized by WHO criteria showed generally similar results. Moreover, there were significant differences in the outcomes of low-birth weight infant and induction of labour across the baseline BMI groups (P = 0.026). (Table 3)

**Table 2:** Correlation between baseline BMI and pregnancy complications, delivery outcome. (classified according to the “Guideline of controlling and prevention on overweight and obesity in China”)^16^.

	Baseline BMI(kg/m^2^)				P value
	BMI < 18.5 kg/m^2^ (n = 140)	18.5 ≤ BMI < 24.0 kg/m^2^ (n = 650)	24.0 ≤ BMI < 28.0 kg/m^2^ (n = 150)	BMI ≥ 28.0 kg/m^2^ (n = 39)	
**Weight gain in the second trimester (kg)**	**9.6 ± 2.9**	**9.3 ± 2.9**	**8.4 ± 2.8**	**7.4 ± 3.6**	**<0.001**
Weight gain in the third trimester (kg)	6.1 ± 2.5	6.2 ± 2.6	5.9 ± 2.8	5.4 ± 2.8	0.142
**GWG (kg)**	**15.7 ± 4.0**	**15.5 ± 4.3**	**14.1 ± 4.5**	**13.0 ± 4.7**	<**0.001**
**Neonate weight (kg)**	**3.23 ± 0.42**	**3.33** ± **0.51**	**3.40 ± 0.58**	**3.26 ± 0.76**	**0.002**
Abortion (n(%))	18.6(26)	14.5(94)	16.7(25)	12.8(5)	0.592
Induction of labour (n(%))	0(0)	0.3(2)	0.7(1)	2.6(1)	0.143
Neonatal death (n(%))	1.4(2)	0.6(4)	0.7(1)	0.0(0)	0.710
Stillbirth (n(%))	0.7(1)	0.2(1)	0.7(1)	0.0(0)	0.569
Preterm birth (n(%))	4.3(10)	4.5(10)	6.8(11)	7.7(17)	0.557
Abnormality of umbilical cord (n(%))	30.0(42)	34.2(222)	35.3(53)	33.3(13)	0.777
Abnormality of amniotic fluid (n(%))	12.1(17)	13.2(86)	16.0(24)	23.1(9)	0.272
Abnormality of placenta (n(%))	0.7(1)	0.5(3)	2.0(3)	0.0(0)	0.225
	**BMI < 18.5 kg/m** ^**2**^ **(n = 140)**	**18.5 ≤ BMI < 24.0 kg/m** ^**2**^ **(n = 650)**	**24.0 ≤ BMI < 28.0 kg/m** ^**2**^ **(n = 150)**	**BMI ≥ 28.0 kg/m2 (n = 39)**	**P value**
Dystocia (n(%))	0.7(1)	1.2(8)	1.3(2)	2.6(1)	0.827
Puerperal infection (n(%))	0.0(0)	1.2(8)	2.7(4)	0.0(0)	0.188
Foetal distress (n(%))	12.9(18)	10.4(67)	10.8(16)	12.8(5)	0.817
**Macrosomia (n(%))**	**2.9(4)**	**7.6(49)**	**10.1(15)**	**15.4(6)**	**0.029**
Low-birth weight infant (n(%))	2.9(4)	4.3(28)	4.7(7)	12.8(5)	0.068
Foetal anomaly (n(%))	1.4(2)	0.6(4)	2.0(3)	0.0(0)	0.342
**GDM (n(%))**	**10.0(14)**	**12.0(78)**	**19.3(29)**	**23.1(9)**	**0.017**
**Gestational hypertension (n(%))**	**2.9(4)**	**3.8(25)**	**9.3(14)**	**5.4(6)**	<**0.001**
Preeclampsia/eclampsia (n(%))	0.7(1)	2.5(16)	0.7(1)	2.6(1)	0.335

**Table 3:** Correlation between baseline BMI and pregnancy complications, delivery outcome. (classified according to the WHO definition)^14^.

	Baseline BMI(kg/m^2^)				P value
	BMI <18.5 kg/m^2^ (n = 140)	18.5 ≤ BMI <24.9 kg/m^2^ (n = 711)	25.0 ≤ BMI <29.9 kg/m^2^ (n = 112)	BMI ≥ 30.0 kg/m^2^ (n = 16)	
**Weight gain in the second trimester (kg)**	**9.6 ± 2.9**	**9.2 ± 2.9**	**8.2 ± 2.9**	**6.8 ± 3.3**	<**0.001**
Weight gain in the third trimester (kg)	6.1 ± 2.5	6.2 ± 2.6	5.7 ± 2.8	4.8 ± 2.7	0.072
**GWG (kg)**	**15.7 ± 4.0**	**15.4 ± 4.4**	**13.9 ± 4.4**	**11.8 ± 3.2**	<**0.001**
**Neonate weight (kg)**	**3.23 ± 0.42**	**3.34 ± 0.52**	**3.33 ± 0.57**	**3.35 ± 1.04**	**0.020**
Abortion (n(%))	18.6(26)	15.2(108)	13.4(15)	6.3(1)	0.478
**Induction of labour (n(%))**	**0(0)**	**0.4(3)**	**0.0(0)**	**6.3(1)**	**0.002**
Neonatal death (n(%))	1.4(2)	0.7(5)	0.0(0)	0.0(0)	0.588
Stillbirth (n(%))	0.7(1)	0.3(2)	0(0)	0.0(0)	0.760
Preterm birth (n(%))	4.3(6)	4.7(33)	6.3(7)	12.5(2)	0.461
Abnormality of umbilical cord (n(%))	30.0(42)	34.2(243)	37.5(42)	18.8(3)	0.354
Abnormality of amniotic fluid (n(%))	12.1(17)	13.6(97)	18.8(21)	6.3(1)	0.336
Abnormality of placenta (n(%))	0.7(1)	0.6(4)	1.8(2)	0.0(0)	0.541
	**BMI <18.5 kg/m2 (n = 140)**	**18.5 ≤ BMI <24.9 kg/m** ^**2**^ **(n = 711)**	**25.0 ≤ BMI <29.9 kg/m** ^**2**^ **(n = 112)**	**BMI ≥ 30.0 kg/m2 (n = 16)**	**P value**
Dystocia (n(%))	0.7(1)	1.3(9)	1.8(2)	0.0(0)	0.849
Puerperal infection (n(%))	0.0(0)	1.3(9)	2.7(3)	0.0(40)	0.273
Foetal distress (n(%))	12.9(18)	10.5(74)	10.7(12)	12.5(2)	0.858
**Macrosomia (n(%))**	**2.9(4)**	**8.1(57)**	**8.0(9)**	**25.0(4)**	**0.009**
**Low-birth weight infant (n(%))**	**2.9(4)**	**4.2(30)**	**6.3(7)**	**18.8(3)**	**0.026**
Foetal anomaly (n(%))	1.4(2)	1.0(7)	0(0)	0.0(0)	0.655
**GDM(n(%))**	**10.0(14)**	**12.7(90)**	**17.9(20)**	**37.5(6)**	**0.008**
**Gestational hypertension (n(%))**	**2.9(4)**	**4.1(29)**	**11.6(13)**	**18.8(3)**	<**0.001**
Preeclampsia/eclampsia (n(%))	0.7(1)	2.3(16)	0.9(1)	6.3(1)	0.299

**Figure 1:**
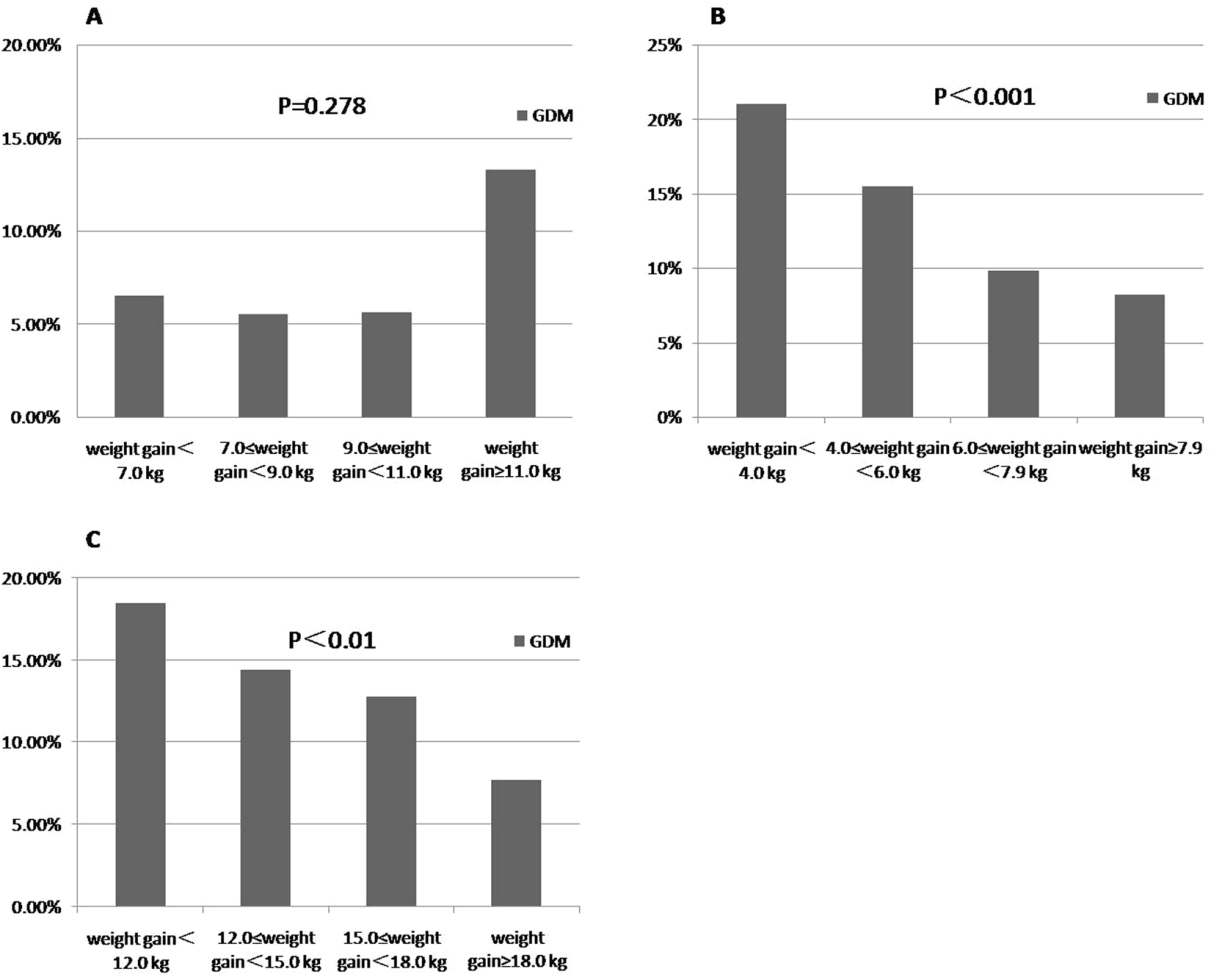
Correlations between weight gain in different trimesters and GDM. (**A**) correlation between weight gain in the second trimester and GDM, (**B**) correlation between weight gain in the third trimester and GDM, (**C**) correlation between gestational weight gain and GDM. GDM, gestational diabetes mellitus.

Analysis on correlation between weight gain in the second trimester and pregnancy complications or delivery outcomes showed that weight gain in the second trimester was significantly and positively correlated with incident of macrosomia (P = 0.002). No significant association was found between weight gain in the second trimester and accessory abnormality, obstetric complications, or gestational complications. (Table 4) (Fig. 1A)

**Table 4:** Correlation between weight gain in the second trimester and pregnancy complications, delivery outcome.

	weight gain in the second trimester (kg)				P value
	weight gain <7.0 (n = 148)	7.0 ≤ weight gain <9.0(n = 222)	9.0 ≤ weight gain <11.0(n = 210)	weight gain ≥ 11.0 (n = 218)	
Weight gain in the third trimester (kg)	5.4 ± 2.5	6.0 ± 2.4	6.2 ± 2.7	6.6 ± 2.8	0.103
**GWG(kg)**	**10.3 ± 2.9**	**13.7 ± 2.5**	**15.9 ± 2.7**	**19.3 ± 3.4**	**0.002**
Neonate weight (kg)	3.21 ± 0.57	3.27 ± 0.46	3.34 ± 0.49	3.45 ± 0.52	0.603
**Baseline BMI (kg/m** ^**2**^ **)**	**22.7 ± 3.5**	**21.5 ± 3.0**	**21.4 ± 3.0**	**21.2 ± 2.8**	**0.001**
Abortion (n(%))	14.5(25)	14.0(36)	16.3(41)	17.4(46)	0.689
Induction of labour (n(%))	1.2(2)	0.8(2)	0(0)	0(0)	0.161
**Neonatal death (n(%))**	**1.7(3)**	**0(0)**	**0(0)**	**0(0)**	**0.004**
Stillbirth (n(%))	0.6(1)	0(0)	0(0)	0(0)	0.215
Preterm birth (n(%))	7.1(12)	3.9(10)	2.8(7)	6.1(16)	0.138
Abnormality of umbilical cord (n(%))	32.4(56)	34.5(89)	34.3(86)	33.0(87)	0.958
Abnormality of amniotic fluid (n(%))	11.0(19)	14.7(38)	13.9(35)	14.0(37)	0.718
Abnormality of placenta (n(%))	1.7(3)	0.8(2)	0.8(2)	0.0(0)	0.230
Dystocia (n(%))	0.6(1)	1.6(4)	1.2(3)	0.8(2)	0.741
Puerperal infection (n(%))	0.6(1)	2.3(6)	1.2(3)	1.1(3)	0.440
Foetal distress (n(%))	10.0(17)	13.6(35)	11.2(28)	9.1(24)	0.399
	**weight gain <7.0 (n = 148)**	**7.0 ≤ weight gain <9.0(n = 222)**	**9.0 ≤ weight gain <11.0(n = 210)**	**weight gain ≥ 11.0 (n = 218)**	**P value**
**Macrosomia (n(%))**	**6.5(11)**	**5.5(14)**	**5.6(14)**	**13.3(36)**	**0.002**
Low-birth weight infant (n(%))	7.6(13)	4.3(11)	4.4(11)	3.0(8)	0.161
Foetal anomaly (n(%))	1.2(2)	1.2(3)	0.4(1)	0.8(2)	0.769
GDM (n(%))	16.2(28)	12.8(33)	10.0(25)	14.0(37)	0.278
Gestational hypertension (n(%))	4.0(7)	2.7(7)	6.8(17)	6.1(16)	0.139
Preeclampsia/eclampsia (n(%))	2.3(4)	2.3(6)	1.2(3)	2.3(6)	0.765

Incidence of macrosomia in different groups categorized as weight gain in the third trimester quartiles showed a significant difference (P = 0.028). Weight gain in the third trimester was significantly and negatively correlated with incidence of neonatal death (P = 0.021), preterm birth (P < 0.001), low birth-weight infants (P = 0.003), and GDM (P < 0.001). (Fig. 1B) No significant association was found between weight gain in the third trimester and accessory abnormality or obstetric complications. (Table 5)

**Table 5:** Correlation between weight gain in the third trimester and pregnancy complications, delivery outcome.

	weight gain in the third trimester(kg)				P value
	weight gain <4.0 (n = 153)	4.0 ≤ weight gain <6.0 (n = 258)	6.0 ≤ weight gain <7.9 (n = 260)	weight gain ≥ 7.9 (n = 229)	
Weight gain in the third trimester (kg)	8.6 ± 3.0	8.9 ± 2.6	9.0 ± 3.0	9.9 ± 3.2	0.901
GWG (kg)	11.2 ± 3.2	13.6 ± 2.7	15.5 ± 3.1	19.5 ± 4.0	0.151
Neonate weight (kg)	3.24 ± 0.59	3.29 ± 0.47	3.33 ± 0.47	3.43 ± 0.47	0.079
**Baseline BMI**	**22.1 ± 3.7**	**21.6 ± 3.0**	**21.6 ± 3.0**	**21.3 ± 2.7**	<**0.001**
Abortion (n(%))	15.5(28)	16.5(51)	15.0(46)	14.6(39)	0.926
Induction of labour (n(%))	0.6(1)	0(0)	0.3(1)	0.4(1)	0.695
**Neonatal death (n(%))**	**1.1(2)**	**0(0)**	**0(0)**	**0(0)**	**0.021**
Stillbirth (n(%))	—	—	—	—	—
**Preterm birth (n(%))**	**11.0(20)**	**4.5(14)**	**3.9(12)**	**1.5(4)**	<**0.001**
Abnormality of umbilical cord (n(%))	33.1(60)	33.7(104)	33.3(102)	34.0(91)	0.998
Abnormality of amniotic fluid (n(%))	10.5(19)	12.6(39)	13.1(40)	17.9(48)	0.112
Abnormality of placenta (n(%))	0.6(1)	1.0(3)	0.3(1)	0.4(1)	0.707
Dystocia (n(%))	2.2(4)	1.3(4)	0.7(2)	1.5(4)	0.529
Puerperal infection (n(%))	1.1(2)	1.3(4)	1.0(3)	1.9(5)	0.813
Foetal distress (n(%))	7.8(14)	11.3(35)	10.2(31)	11.9(32)	0.520
	**weight gain <4.0 (n = 153)**	**4.0 ≤ weight gain <6.0 (n = 258)**	**6.0 ≤ weight gain <7.9 (n = 260)**	**weight gain ≥ 7.9 (n = 229)**	**P value**
**Macrosomia (n(%))**	**6.1(11)**	**4.9(15)**	**7.9(24)**	**11.3(30)**	**0.028**
**Low-birth weight infant (n(%))**	**8.8(16)**	**3.6(11)**	**3.6(11)**	**1.9(5)**	**0.003**
Foetal anomaly (n(%))	1.7(3)	1.0(3)	1.0(3)	0.4(1)	0.586
**GDM (n(%))**	**21.0(38)**	**15.5(48)**	**9.8(30)**	**8.2(22)**	<**0.001**
Gestational hypertension (n(%))	6.1(11)	3.2(10)	3.9(12)	7.8(21)	0.054
Preeclampsia/eclampsia (n(%))	2.8(5)	1.3(4)	1.6(5)	2.6(7)	0.559

We further analysed the association between gestational weight gain and different pregnancy complications or delivery outcomes. Results showed that gestational weight gain was significantly and positively correlated with incidence of macrosomia (P = 0.002) and negatively correlated with the neonatal death (P < 0.001), stillbirth (P = 0.007), preterm birth (P < 0.001), infants of low birth weight (P = 0.001), and GDM (P = 0.009). (Fig. 1C) (Table 6)

**Table 6:** Correlation between gestational weight gain and pregnancy complications, delivery outcome.

	GWG (kg)				P value
	weight gain <12.0 (n = 162)	12.0 ≤ weight gain <15.0 (n = 226)	15.0 ≤ weight gain <18.0 (n = 221)	weight gain ≥ 18.0 (n = 211)	
**Weight gain in the second trimester (kg)**	**5.8 ± 2.0**	**8.3 ± 1.7**	**9.6 ± 1.8**	**12.1 ± 2.6**	**0.001**
**Weight gain in the third trimester (kg)**	**3.8 ± 1.9**	**4.9 ± 1.6**	**6.5 ± 1.8**	**8.7 ± 2.5**	<**0.001**
Neonate weight (kg)	3.16 ± 0.65	3.30 ± 0.49	3.34 ± 0.48	3.46 ± 0.49	0.061
**Baseline BMI (kg/m** ^**2**^ **)**	**22.7 ± 3.5**	**21.5 ± 3.0**	**21.4 ± 3.0**	**21.3 ± 2.7**	<**0.001**
Abortion (n(%))	16.9 (33)	12.1 (31)	19.0 (52)	14.2 (35)	0.137
Induction of labour (n(%))	1.5 (3)	0.4 (1)	0.0 (0)	0.4 (1)	0.136
**Neonatal death (n(%))**	**3.1 (6)**	**0.0 (0)**	**0.0 (0)**	**0.0 (0)**	<**0.001**
**Stillbirth (n(%))**	**1.5 (3)**	**0.0 (0)**	**0.0 (0)**	**0.0 (0)**	**0.007**
**Preterm birth (n(%))**	**11.6 (22)**	**3.5 (9)**	**2.6 (7)**	**3.7 (9)**	<**0.001**
Abnormality of umbilical cord (n(%))	34.9 (68)	36.6 (94)	30.8 (84)	34.1 (84)	0.552
Abnormality of amniotic fluid (n(%))	11.3 (22)	13.2 (34)	14.3 (39)	16.7 (41)	0.425
Abnormality of placenta (n(%))	1.5 (3)	0.8 (2)	0.7 (2)	0.0 (0)	0.306
Dystocia (n(%))	0.5 (1)	1.6 (4)	0.4 (1)	1.6 (4)	0.355
	**weight gain < 12.0 (n = 162)**	**12.0 ≤ weight gain <15.0 (n = 226)**	**15.0 ≤ weight gain <18.0 (n = 221)**	**weight gain ≥ 18.0 (n = 211)**	**P value**
Puerperal infection (n(%))	1.0 (2)	1.6 (4)	1.1 (3)	1.6 (4)	0.917
Foetal distress (n(%))	11.7 (22)	9.0 (23)	13.2 (36)	10.2 (25)	0.446
**Macrosomia (n(%))**	**5.3 (10)**	**5.9 (15)**	**6.6 (18)**	**13.5 (33)**	**0.002**
**Low-birth weight infant (n(%))**	**10.0 (19)**	**3.9 (10)**	**3.7 (10)**	**2.5 (6)**	**0.001**
Foetal anomaly (n(%))	2.6 (5)	0.4 (1)	0.7 (2)	0.8 (2)	0.117
**GDM (n(%))**	**18.5 (36)**	**14.4 (37)**	**12.8 (35)**	**7.7 (19)**	**0.009**
Gestational hypertension (n(%))	3.1 (6)	5.4 (14)	5.1 (14)	6.1 (15)	0.525
Preeclampsia/eclampsia (n(%))	2.6 (6)	1.9 (5)	1.1 (3)	2.4 (6)	0.632

### Multivariate regression analysis on the association between baseline BMI, weight gain and pregnancy complications or delivery outcomes

After adjusting for confounding factors, backwards stepwise multivariate regression model showed that baseline BMI was the independent risk factor for the development of gestational hypertension and macrosomia. Weight gain in the second trimester was the independent risk factor for the development of macrosomia. Gestational weight gain was the independent risk factor for the development of macrosomia. The odds ratio, 95% confidence interval (CI) and P value for each model are shown in Table 7.

**Table 7:** Multivariable regression analysis on the association between baseline BMI, weight gain and pregnancy complications, delivery outcome.

	**OR**	**95% CI**	**P value**
**Baseline BMI**			
Macrosomia	1.131	1.035–1.236	0.006
Gestational hypertension	1.156	1.044–1.279	0.005
**Weight gain in the second trimester**			
Macrosomia	1.145	1.027–1.276	0.015
**Weight gain in the third trimester**			
Preterm birth	0.770	0.646–0.916	0.003
Low-birth weight infant	0.813	0.668–0.990	0.028
GDM	0.828	0.743–0.923	0.001
**Gestational weight gain**			
Macrosomia	1.096	1.020–1.178	0.013
Low-birth weight infant	0.890	0.793–0.998	0.045
GDM	0.922	0.865–0.982	0.011

## Discussion

The present study showed a correlation between baseline BMI, pregnancy weight gain and gestational complications, adverse pregnancy outcomes, and status of neonate in varying degrees. Baseline BMI, weight gain in the second trimester and gestational weight gain were significantly, positively associated with macrosomia and its independent risk factor. Baseline BMI was also positively associated with GDM. Weight gain in the third trimester and gestational weight gain were significantly, negatively associated with development of GDM, preterm birth, low birth weight infant and neonatal death.

In 2009, the Institute of Medicine in America re-examined the guideline on weight gain during pregnancy. The recommended range of gestational weight gain for women who are underweight (BMI < 18.5 kg/m^2^), normal weight (BMI ranges from 18.5 to 24.9 kg/m^2^), overweight (BMI ranges from 25 to 29.9 kg/m^2^), and obese (BMI 30 ≥ kg/m^2^) was 12.5–18, 11.5–16, 7–11.5, 5–9 kg, respectively. In China, we have been following the recommendation of IOM and there is no established recommendation for Chinese women. Results from our study showed that the average gestational weight gain for included population was 15.3 kg. Gestational weight gain for women who were overweight and obese exceeded the recommended weight gain. This result partly reflected the current status of Chinese pregnant women. Analysis based on baseline BMI categorized by Chinese BMI criteria and WHO criteria showed generally similar results. However, there were differences in results of induction of labour and low-birth weight infant, which suggest that subgroup analysis were needed for establishing recommendations in Chinese women.

Results from this study showed that the morbidity of GDM and gestational hypertension was positively correlated with baseline BMI. In multivariable regression analysis, baseline BMI was found to be the independent risk factor of macrosomia and gestational hypertension. These findings are consistent with results of previous studies. A large cohort study including 4,312 women comparing pre-pregnancy BMI categories with obstetrical and neonatal outcomes in Canada showed that compared to women with normal BMIs, overweight, obese, and morbidly obese women had an increased risk of preeclampsia, gestational hypertension, GDM, and macrosomia^3^. Meta-analysis also indicated that high maternal weight is associated with a substantially higher risk of GDM^5^. Thus, strategies aimed at preventing obesity in young women and keeping appropriate preconception weight in pre-pregnant women are essential for the prevention of gestational complications.

Our study showed potential correlations between neonatal death, stillbirth and gestational weigh gain in different trimesters and the overall GWG. However, neonatal death and stillbirth only occurred in the lowest weigh gain group, the small number of cases likely limited statistical power. Opinions from the Institute of Medicine in America suggested that for women who were underweight, normal weight, overweight, and obese, recommended rates of weight gain in the second and third trimesters were 0.44–0.58 kg, 0.35–0.50 kg, 0.23–0.33 kg, 0.17–0.27 kg per week, respectively. In China, there are still no established recommendations for appropriate weight gain in the second and third trimester. Results from our study suggested that disproportionately low and excessive weight gain in the second and third trimester were associated with adverse obstetric outcomes, obstetric complications, abnormality of foetus, and gestational complications. Studies with larger sample sizes are needed to explore the appropriate weight gain to achieve best pregnancy outcomes.

However, GDM was shown to be negatively associated with weight gain during pregnancy. In multivariable regression analysis, weight gain in the third trimester and gestational weight gain were negatively associated with incidence of GDM. The potential reasons for this finding are as follows: the diagnosed test was done at 24^th^–28^th^ week during the second trimester, pregnant women who have been diagnosed GDM may receive more advice about weight control and paid more attention to diet and exercise during the third trimester. As excessive weight gain might has already happened before the test, positive correlation was found between the second trimester weight gain and macrosomia. In addition, the metabolic disorder induced by GDM might influence weight gain during pregnancy.

In this retrospective study, we analysed the association between baseline BMI, weight gain in different trimesters, gestational weight gain, and pregnancy complications and delivery outcomes. The study has several limitations. The included individuals were women with complete prenatal care data who had better compliance and made more effort in weight control compared with those pregnant women with worse compliance, which may cause selection bias in the study. Our study is designed as a single-centre, cross-sectional study. Pregnant women delivered in Peking University People’s Hospital might have unknown conditions and complications; thus, there is selection bias in this study. Moreover, BMI at baseline was calculated according to the weight at 12^th^ week during pregnancy and did not indicate the pre-pregnancy status objectively.

## Conclusions

There is correlation between baseline BMI, pregnancy weight gain, and gestational complications, including adverse pregnancy and status of neonate in varying degrees. Maintaining appropriate pre-pregnancy weight and gestational weight gain is essential in the prevention of pregnancy complications and prognosis of maternal and infant.
